# Plastin 3 Expression Does Not Modify Spinal Muscular Atrophy Severity in the ∆7 SMA Mouse

**DOI:** 10.1371/journal.pone.0132364

**Published:** 2015-07-02

**Authors:** Vicki L. McGovern, Aurélie Massoni-Laporte, Xueyong Wang, Thanh T. Le, Hao T. Le, Christine E. Beattie, Mark M. Rich, Arthur H. M. Burghes

**Affiliations:** 1 Department of Molecular and Cellular Biochemistry, The Ohio State University Wexner Medical Center, Columbus, Ohio, United States of America; 2 Department of Neuroscience, Cell Biology, and Physiology, Wright State University, Dayton, Ohio, United States of America; 3 Department of Neuroscience, The Ohio State University Wexner Medical Center, Columbus, Ohio, United States of America; 4 Department of Neurology, The Ohio State University Wexner Medical Center, Columbus, Ohio, United States of America; Children's Hospital of Pittsburgh, University of Pittsburgh Medical Center, UNITED STATES

## Abstract

Spinal muscular atrophy is caused by loss of the *SMN1* gene and retention of *SMN2*. The *SMN2* copy number inversely correlates with phenotypic severity and is a modifier of disease outcome. The *SMN2* gene essentially differs from *SMN1* by a single nucleotide in exon 7 that modulates the incorporation of exon 7 into the final SMN transcript. The majority of the *SMN2* transcripts lack exon 7 and this leads to a SMN protein that does not effectively oligomerize and is rapidly degraded. However the *SMN2* gene does produce some full-length SMN and the *SMN2* copy number along with how much full-length SMN the *SMN2* gene makes correlates with severity of the SMA phenotype. However there are a number of discordant SMA siblings that have identical haplotypes and *SMN2* copy number yet one has a milder form of SMA. It has been suggested that *Plastin3* (*PLS3*) acts as a sex specific phenotypic modifier where increased expression of *PLS3* modifies the SMA phenotype in females. To test the effect of *PLS3* overexpression we have over expressed full-length PLS3 in SMA mice. To ensure no disruption of functionality or post-translational processing of PLS3 we did not place a tag on the protein. PLS3 protein was expressed under the Prion promoter as we have shown previously that SMN expression under this promoter can rescue SMA mice. High levels of *PLS3* mRNA were expressed in motor neurons along with an increased level of PLS3 protein in total spinal cord, yet there was no significant beneficial effect on the phenotype of SMA mice. Specifically, neither survival nor the fundamental electrophysiological aspects of the neuromuscular junction were improved upon overexpression of *PLS3* in neurons.

## Introduction

Proximal Spinal Muscular Atrophy (SMA) is an autosomal recessive disorder and the leading genetic cause of infant mortality [1, 2]. The disorder is characterized by loss of alpha motor neurons in anterior spinal cord and atrophy of muscle [3]. SMA is caused by loss of the *Survival Motor Neuron 1* gene (*SMN1*) and retention of *SMN2* [4, 5]. The *SMN1* and *SMN2* genes essentially differ at a single nucleotide within exon 7 that results in disruption of a splice modulator and the majority of the *SMN2* transcript lacking exon 7 [6–10]. The SMN lacking exon 7 encoded amino acids does not efficiently oligomerize and thus is rapidly degraded [11, 12]. This leads to low SMN levels in SMA [13, 14]. The major modulator of the SMA phenotype is the *SMN2* gene itself as it does produce some full-length SMN protein. Specifically, there is an inverse correlation of *SMN2* copy number to phenotypic severity where mild type III SMA cases have more copies of *SMN2* than severe type 1 cases [15, 16]. Furthermore, certain *SMN2* alleles, namely the variant 859G>C, alters the incorporation of SMN exon 7 and thus results in greater SMN production [17, 18]. Indeed, a study of Spanish SMA patients showed that type 1 patients do not have this variant, type 2 patients (with two copies of *SMN2)* are heterozygous for this variant, and type 3b patients (with two copies of *SMN2*) are homozygous for this variant [19]. Thus there is a clear relationship between the amount of full-length SMN that can be produced by a particular genotype and the severity of SMA. However there are some discrepancies to the *SMN2* copy number rule. In particular, there are reports on a series of families where siblings with identical haplotypes, including *SMN2* copy number, have markedly different phenotypes [15, 20–25]. While this may be more common in type 2 and 3 cases [20, 22, 23] siblings with discordant phenotypes also occur with type 1 SMA [24, 26–28]. Furthermore, there appears to be a gradation of phenotypic severity where type 1 and type 2 SMA occurs in the same family, or type 2 and type 3 SMA, or type 3 SMA and unaffected siblings. This strongly implies that the modifier of SMA phenotype can alter all SMA types in much the same way that *SMN2* copy number alters SMN levels and SMA.

There are two major paths that can be considered for modification of SMA phenotype in discordant siblings. First, there could be alteration in a factor that acts on the *SMN2* loci to influence the amount of full-length SMN produced by *SMN2*. In this regard, many proteins have been reported to bind SMN exon 7 and the surrounding introns to regulate the incorporation of exon 7 [29, 30]. The second possibility involves modifiers of the SMA phenotype that do not alter SMN levels. SMN has been shown to function in the assembly of Sm proteins onto snRNA to form SnRNPs and has been suggested to play a role in a series of other assembly reactions [31]. SMN has also been proposed to have other functional roles particularly in the axon. Indeed knockdown of Smn in zebrafish results in axonal abnormalities and motor neurons cultured from severe mice have been reported to have reduced transport of ß-actin to the growth cone [32, 33]. However there is no defect of axon out growth *in vivo* in the SMA mouse embryo [34]. Genes that could influence these SMN functions or their outcome might act as modifiers of the SMA phenotype.

Studying discordant families has produced reports of potential modifiers of SMA. Originally it was reported that SMN levels were elevated in fibroblast cultures of milder cases in discordant families [35]. Subsequently the levels were reported to be the same [36] at least in lymphoblasts. Analysis of the expression changes that occur in lymphoblasts revealed that PLS3 (PLS3, T-Plastin, or T-fimbrin; MIM 300131, Xq23) had elevated levels in some siblings with discordant phenotypes. The authors concluded that PLS3 acts as a female specific modifier of SMA. All the sib pairs except one contained a male and female where the female was asymptomatic. Thus, even if expression of PLS3 were found in the male patients it would not improve the phenotype. As such it is difficult to interpret the significance of this observation based on a single female sib pair. In a second study no association of PLS3 expression was found in discordant female sib pairs [37]. In fact, PLS3 expression was slightly increased in the affected female sibling and not the asymptomatic individual. Recently, a tagged form of PLS3 protein has been investigated in SMA mice [37]. The authors report some mild benefits to the SMA phenotype under certain conditions. However, PLS3 expression is highly modulated at the protein level and the placement of a tag can affect both function and protein turnover [37]. We thus have investigated whether the overexpression of PLS3 without a tag can modify the SMA phenotype in mice.

In order to determine if *PLS3* acts as a modifier of the SMA phenotype we generated transgenic mice expressing human *PLS3* under control of the Prion (PrP) promoter. We have shown previously that the PrP:SMN transgene resulted in high expression of SMN in all neurons completely rescued the SMA phenotype in the mouse [38]. We proposed that if *PLS3* is a SMA modifier then high expression of human *PLS3* under control of this same promoter should alter survival and phenotype of Δ7 SMA mice. We found no increase in weight or survival of PrP:PLS3, Δ7 SMA mice in three different transgenic lines. Furthermore, we found no improvement in neuromuscular junction physiology in these PrP:PLS3 Δ7 SMA mice. Our results indicate that *PLS3* is not a viable therapeutic target to modify the SMA phenotype in humans.

## Materials and Methods

### Ethics statement

This study was carried out in strict accordance with the recommendations in the Guide for the Care and Use of Laboratory Animals of the University Laboratory Animal Resources at The Ohio State University and Wright State University. Our protocol was approved by The Ohio State University Institutional Animal Care and Use Committee (IACUC), Office of Responsible Research Practices, under Permit Number 2008A0089. Anesthesia was administered with Isoflurane according to our animal protocol. Carbon Dioxide followed by cervical dislocation for secondary means of confirmation was used for euthanasia according to our approved protocol.

### Generation of *PLS*3 expressing transgenes

Human *PLS3* cDNA (Clone ID 6064540, Open Biosystems) was end filled and cloned into the Prion (PrP) vector that contains the mouse prion promoter and exon 1, intron 1, part of exon 2 [34]. Human *PLS3* cDNA was directionally cloned between the end filled *KpnI* and *XhoI/SalI* sites in PrP vector exon 2. The resulting construct was sequenced, linearized by digestion with *PvuI*, gel purified and dialyzed. The PrP:PLS3 plasmid was transfected into MN1 cells using Lipofectamine 2000 Transfection Reagent according to the manufacture’s instructions (Invitrogen). After expression PLS3 was confirmed by Western blot the construct was injected into fertilized FVB/N Δ7 (JAX 5025) mouse oocytes to generate transgenic mice. The PrP:PLS3 transgene was detected with PrP exon 2 FP 5`GGACTCGTGAGTATATTTCAG and PLS3 RP 5`GAAGGTCTTGGCAATATCACTACT. Three founder mice, named PLS-14, PLS-39 and PLS-46, were bred to Δ7 mice (*SMN2* ^*+/+*^; *Smn* ^*+/-*^: Δ7SMN^+/+^). PLS-14 female founder would not breed therefore an ovary transfer was performed into a FVB/N female and then progeny were bred to Δ7 mice. The SMN2 transgene and mouse knockout allele were detected as previously described [38, 39]. PLS-39 and PLS-46 lines were bred to homozygosity, PLS-14 is not homozygous viable. Each transgenic line conformed to Mendelian autosomal patterns of inheritance thus multiple transgenic insertion sites were not detected. Homozygosity of the transgene was determined by qPCR on genomic tail DNA.

### Weight and survival measurements

Mice were housed and fed at no more than 5 per cage according to our IACUC approved animal protocol and the Standard Operating Procedure for SMA mice SMA_M.2.2.003 (Treat-NMD.eu). Weight and survival analysis was performed as previously described for the Δ7 line [40, 41]. A similar number of male and female mice were observed and weighed at minimum once per day from the date of birth (P0) until death or day 21 (P21). Any change in behavior, appearance or survival was noted. Mice where humanely euthanized when they achieved exclusion criteria including the inability of neonatal SMA mice to go to the mother (homing) to suckle and loss of greater than 20% of maximum weight that particular animal achieved according to our IACUC approved animal protocol. Required steps were taken to minimize suffering of the mice including administration of systemic analgesia (Motrin) in the water bottle at 100mg/5ml providing a dose of approximately 30 mg/kg when needed. All animals were grouped according to genotype. Kaplan-Meier survival curves and mean weights were graphed with SigmaPlot.

### Expression of *PLS3* in Brain and Spinal cord of PrP:PLS3 SMA mice

RNA was isolated from brain and total spinal cord at P12 using TRIzol reagent (Invitrogen), purified with the RNeasy kit (Qiagen) and converted to cDNA as previously described [38]. Primers used to detect cDNA include: PrP:PLS3 transgene, FP: 5`CGGATCAGCAGACCGATTCT, RP:5`GCACCTCGGAATCTTTGCA, probe: FAM-ATCGGTGGCAGGACT-MGB; mouse Pls3, FP:5`CCGCGACTCCCTATGAATCTT (mouse specific), RP:5`GAGTTCATCAAGCTCATCTTTGGA, Probe:FAM-ACATGGATGAGATGGC-MGB; Mouse cyclophilin, FP:5`GTCAACCCCACCGTGTTCTT, RP: 5`TTGGAACTTTGTCTGCAAACA, Probe: VIC-CTTGGGCCGCGTCT-MGB..

Reactions were run on the ABI 7300 Real-Time PCR System. Relative human and mouse *PLS3* levels were determined by normalizing to mouse cyclophilin (PIPB) expression. Three technical replicates and five to seven biological replicates were performed for each sample. All three transgenic lines as well as a non-transgenic control were tested.

### Expression of *PLS3* in LCM isolated motor neurons of PrP:PLS3 SMA mice

Motor neurons were collected from fresh frozen spinal cord sections on a Zeiss Palm Robo 3 Laser Capture Microdissection System. Motor neurons were located based on size and location in the anterior horn after Nissl staining for contrast. RNA was isolated with the Ambion RNA-aqueous Micro kit (AM1931) and aRNA was generated with the Arcturus PicoPure RNA Isolation Kit (ABI KIT0204). Droplet generation and reader analysis were performed on the QX200 (Bio-Rad). 15,000 to 18,000 droplets containing cDNA, primers, probe, 2x ddPCR SuperMix for Probes, and droplet generation oil were generated and amplified. Primers used to detect cDNA include: Prion:PLS3 transgene, FP: 5`CGGATCAGCAGACCGATTCT, RP:5`GCACCTCGGAATCTTTGCA,probe:FAM-ATCGGTGGCAGGACT-MGB; mouse Pls3, FP:5`ccgcgactccctatgaatctt (mouse specific), RP:5`gagttcatcaagctcatctttgga, Probe:FAM-ACATGGATGAGATGGC-MGB; Mouse cyclophilin, FP:5`GTCAACCCCACCGTGTTCTT, RP: 5`TTGGAACTTTGTCTGCAAACA, PROBE: VIC-CTTGGGCCGCGTCT-MGB. A sufficient number of positive and negative droplets were read by the QX200 reader and quantified using the QuantaSoft software (Bio-Rad). The concentration of transcripts was determined using Poisson statistical distributions and relative human or mouse plastin levels were determined by normalizing to mouse cyclophilin expression. Two technical replicates (for a total of >20,000 droplet PCR reactions) and three biological replicates were performed for each sample. All three transgenic lines as well as a non-transgenic control were tested.

### Protein expression of PLS3 spinal cord

Brain and spinal cord tissues were harvested from 3 PrP:PLS3 male mice at P10 for each transgenic line and non-transgenic controls. Protein isolation and western blots were performed as previously described [42]. The antibody used to detect PLS3 (1:250, GenTEX, 103323) is not specific for human PLS3 thus the total amount of mouse and human PLS3 protein was detected. Accurate size detection of the PLS3 protein was confirmed by detecting PrP:PLS3 protein isolated from transfected MN-1 cells. Blots were incubated with anti-rabbit Fab fragment HRP (1:10,000, Jackson ImmunoResarch, 111-035-006). Three concentrations of the same protein sample were loaded on the gel (90μg, 45μg and 25μg). Blots were probed with mouse anti beta-tubulin mAb (1:10,000, Abcam Ab7291) to measure protein loading and developed using the ECL system as described by the manufacturer (GE Healthcare Life Sciences). Blots where scanned and quantified as described (http://lukemiller.org/index.php/2010/11/analyzing-gels-and-western-blots-with-image-j/) and the area under each peak determined with ImageJ software. Statistical analysis was performed with SigmaPlot. All samples collected for RNA and protein analysis were from male mice.

### Zebrafish axon correction

Zebrafish embryos were maintained at ~28.5°C and staged by hours post fertilization (hpf) [43]. Transgenic *Tg(mnx1*:*0*.*6hsp70*:*GFP)os26* [44] embryos expressing GFP in their motor axons (referred to as *Tg(mnx1*:*GFP)* embryos) were used for *smn* morpholino (MO) and human *PLS3* mRNA injections. The antisense *smn* MO was described previously by McWhorter et al. [33]. One cell-stage embryos were injected with 9ng of *smn* MO with or without 250 pg of synthetic human *PLS3* mRNA.

To generate mRNA, human *PLS3* in pCMV.sport 6 vector was subcloned into pCS2+ vector and linearized with *Not*I. Capped RNA was generated using the Sp6 mMESSAGE mMACHINE kit (Ambion, Austin, TX) following the manufacturer’s instructions.

To visualize motor axons, 28 hpf *Tg(mnx1*:*GFP)* embryos were anesthetized with tricaine (160 μg/ml) and fixed overnight at 4°C in 4% formaldehyde/PBS. After removing from fix, embryos were mounted on glass coverslips for observation under a Zeiss Axioplan microscope, scored [45] and imaged on a Leica confocal microscope. Ten motor axons were scored per animal and animals were designated as containing severe, moderate, mild, or no defects based on criteria in Carrel et al., [45]. Three separate experiments were performed and for each condition (control, *smn* MO and *smn* MO + *PLS3* RNA), n was between 19–24 embryos. Data was plotted as mean ± SEM for the three experiments and Mann-Whitney non-parametric rank test was used to test significance.

### Electrophysiological recording from neuromuscular junctions (NMJs)

Physiology was performed on P10-P11 mouse NMJs from the tibialis anterior muscle as previously described [46]. Briefly, muscle was perfused with Ringer solution containing (in millimoles per liter): NaCl, 118; KCl, 3.5; CaCl2, 2; MgSO4, 0.7; NaHCO3, 26.2; NaH2PO4, 1.7; glucose, 5.5 (pH 7.3–7.4, 20–22°C) equilibrated with 95%O2 and 5% CO2. All NMJs were imaged by staining with 4-Di-2-ASP and impaled within 100 um of the endplate. Muscle fibers were crushed away from the endplate band and voltage clamped to -45 mV. Quantal content was determined directly by dividing evoked endplate current (EPC) amplitude by the average miniature endplate current (MEPC) amplitude for a given NMJ. Repetitive stimulation was given by applying a 50 Hz train of 10 pulses. Statistics. All data are expressed as mean ± SEM.

### Statistical analysis

Quantitative data are expressed as mean ± SEM. Values for number of animals are given in Results and the figure legends. Kaplan-Meier survival curves were generated with SigmaPlot and statistical significance was determined using the log-rank test. The Holm-Sidak method was used for all pairwise multiple comparisons. Significance of weight data were determined with one-way ANOVA and by the Compare Growth Curve function found in the R-Package (Statmod). Specific tests for qPCR, ddPCR and western blot analysis are as described in the figure legends. Values of p<0.05 were considered significant.

## Results

### Generation of PLS3 expressing transgenes

**Fig 1:**
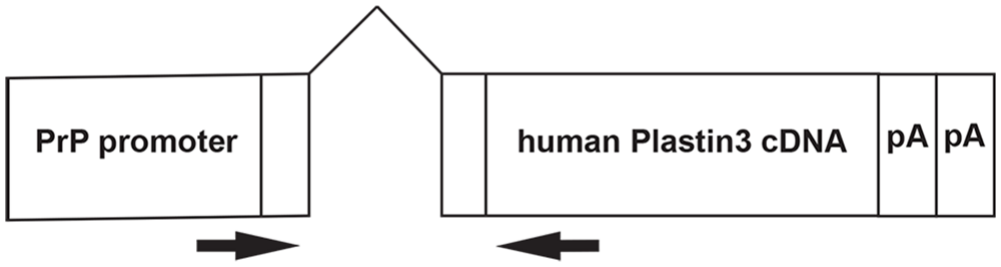
Diagram of the PrP:PLS3 construct. A construct containing the mouse Prion (PrP) promoter, exon 1, intron 1 and part of exon 2 was fused to the human *PLS3* cDNA. This same promoter was used previously to express SMN in neurons [38]. Arrows indicate the location of PrP exon1 forward primer and PLS3 reverse primer used to specifically amplify *PLS3* transcripts produced by this transgene.

We have previously corrected SMA mice using SMN driven by the prion promoter (PrP) [38]. Thus this promoter expresses in the required spatial and temporal pattern in the nervous system. The construct contains human PLS3, without a tag, expressed under the mouse prion promoter. While tagging a protein at the amino terminus is useful in detection of the protein, the tag can alter protein function and/or the decay of the protein [47] [48]. The construct used for generating the transgenes is diagramed in Fig 1. The construct was injected into the pronucleus of fertilized FVB/N Δ7 (JAX 5025) mouse oocytes. A total of 3 expressing lines named PLS-14, PLS-39 and PLS-46 were obtained. The PrP:PLS3 lines were crossed to the Δ7 SMA mouse model to obtain mice that contained the PrP:PLS3 transgene, two copies of *SMN2*, and the SMNΔ7 transgene (PrP:PLS3 ^+/+^;*SMN2* ^*+/+*^; SMNΔ7^+/+^; *Smn* ^*+/-*^). The progeny for each line were interbred to obtain SMA mice containing the PrP:PLS3 transgene (PLS-14^+/-^;*SMN2* ^*+/+*^; SMNΔ7^+/+^; *Smn* ^*-/-*^), (PLS-39^+/+^;*SMN2* ^*+/+*^; SMNΔ7^+/+^; *Smn* ^*-/-*^), (PLS-46^+/+^;*SMN2* ^*+/+*^; SMNΔ7^+/+^; *Smn* ^*-/-*^). Mice that were homozygous for the PLS-14 transgene were not viable.

### Functionality of PLS3 coding sequence used to generate transgenic mice

**Fig 2:**
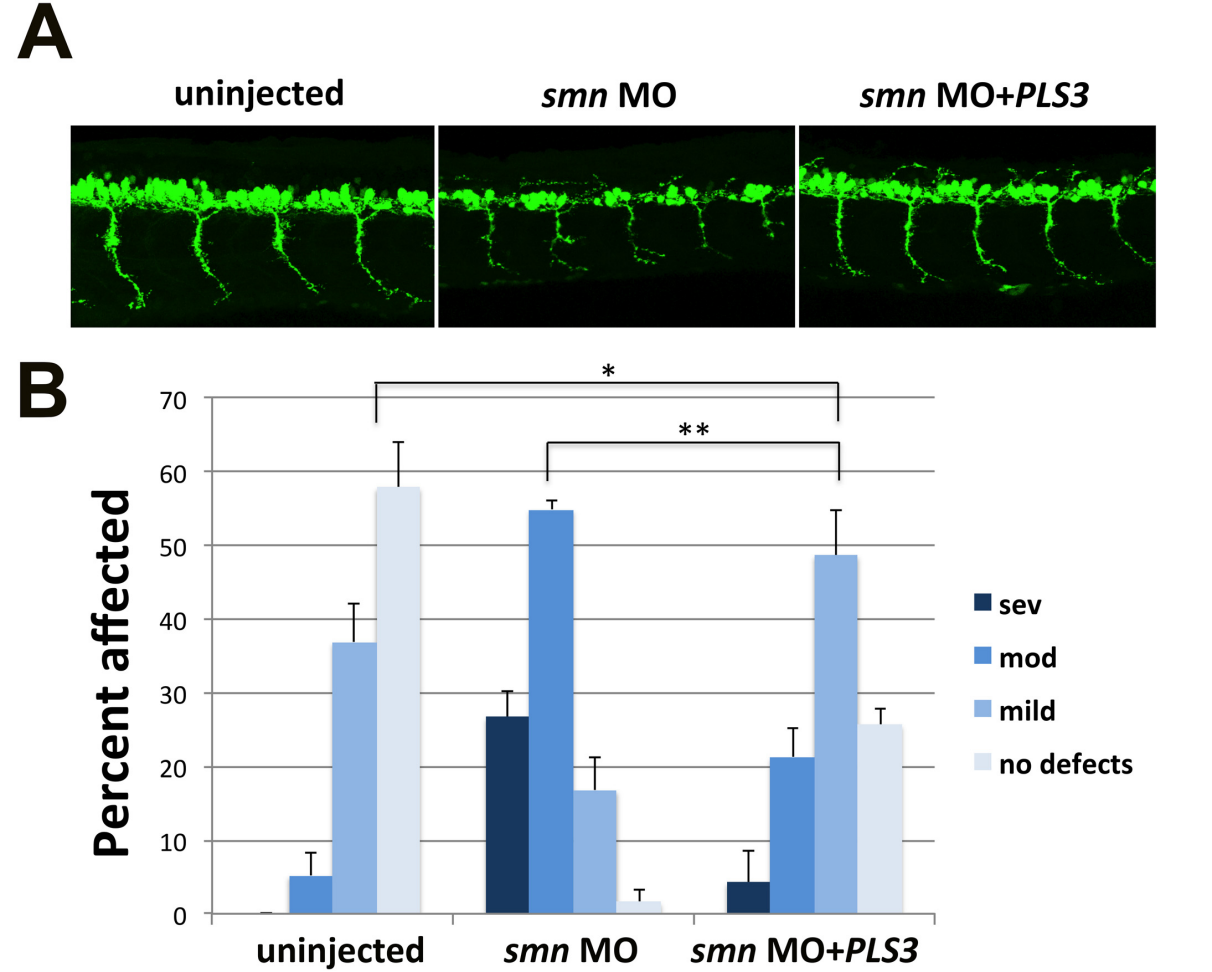
Coding sequence of the *plastin 3* transgene is functional. (A) Lateral view of 28 hpf *Tg(mnx1*:*GFP)* showing ventrally extending motor axons of uninjected, *smn* morpholino (MO) injected, or *smn* MO + *plastin 3* RNA (*PLS3*) injected embryos. (B) Embryos from three separate experiments (n = 19–24 embryos/experiment) were scored as having severe, moderate, mild, or no defects based on criteria in Carrel et al [45]. Mean ± SEM was plotted and significance was determined by two-tailed Mann-Whitney non-parametric rank test. *p = 0.0001, **p<0.0001.

To test whether the *PLS3* cDNA used in generating our transgenic lines encoded functional PLS3 protein, we tested *PLS3* mRNA in zebrafish. Decreasing *smn* transiently in zebrafish embryos using an *smn* morpholino has been shown to result in motor axon defects [33]. Furthermore, injecting human *PLS3* RNA into these *smn* morphants rescued the axonal defects [36, 49]. Therefore, we tested the *PLS3* sequence used to construct our transgene in this same assay and found that it was able to significantly rescue the *smn* morphant motor axon defects (Fig 2). This finding demonstrates that the *PLS3* coding sequence used to generate our transgenic mice produced functional PLS3 protein.

### Expression of *PLS3* in brain and spinal cord tissue

**Fig 3:**
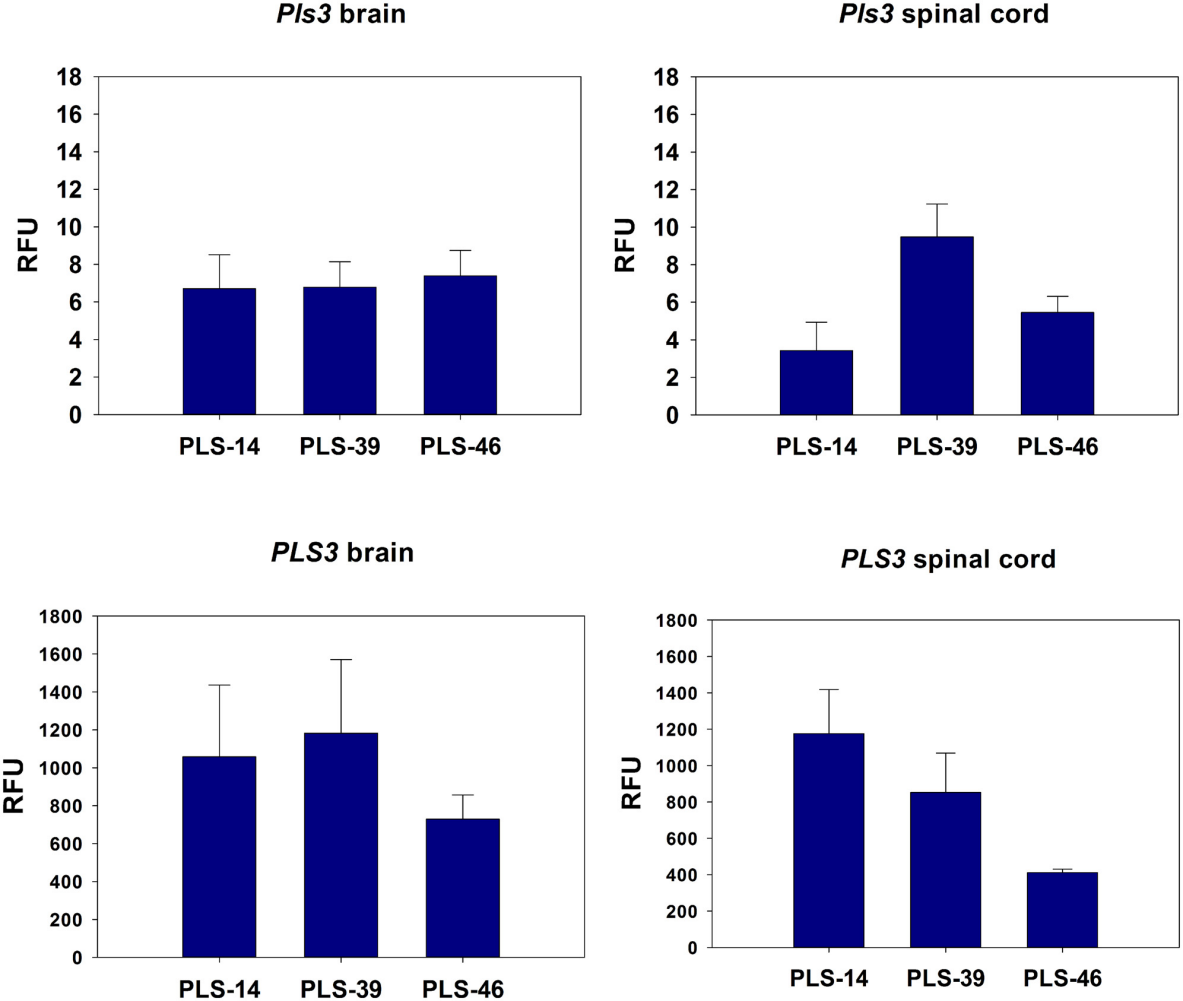
Expression of PrP:PLS3 transcript in brain and spinal cord. (A) Endogenous mouse Pls3 expression in the brain (PLS-14: 6.7±0.8, PLS-39: 6.8±1.4, PLS-46: 7.4±1.4, control: 3.5±0.0 RFU, p = 0.197) and (B) spinal cord (PLS-14: 3.4±0.7, PLS-39: 9.5±0.8, PLS-46: 5.5±0.4, control: 5.4±0.0 RFU, p≤0.7) was measured using quantitative RT-qPCR. The difference in Pls3 expression between the lines was not statistically different from controls (ANOVA). (C) Human PLS3 expression in the brain (PLS-14:1057.9±168.8, p<0.001, PLS-39:1182.0±174.0 p<0.001, PLS-49:728.4±57.6 p = 0.005 vs. control 0±0.0 RFU) and (D) spinal cord (PLS-14: 1175.6±108.5 p<0.001, PLS-39: 852.3±96.7 p<0.001, PLS-46: 410.9±8.9 p<0.01 vs. control 0±0.0 RFU) is also shown for each transgenic line at P10. Note that there is a nearly 100-fold increase in expression of the PrP:PLS3 transgene the levels of endogenous mouse Pls in both brain (A,C) and spinal cord (B,D). (n = 5–7 animals for each transgenic line and tissue assayed). RFU is defined as Relative Fluorescent Units.

To determine expression of Prp:PLS3 in the brain and spinal cord we used quantitative qRT-PCR. Endogenous mouse Pls expression (Fig 3A and 3B) and human PLS3 expression (Fig 3C and 3D) was measured by quantitative RT-qPCR in the brain (Fig 3A and 3C) and in total spinal cord (Fig 3B and 3D) tissue at P10 for each transgenic line (PLS-14, PLS-39 and PLS-46). We found that the expression of the PrP:PLS3 transgene was nearly 100 fold increased over endogenous mouse Pls3 levels in both brain and spinal cord in all three transgenic lines. (n = 5–7 for each transgenic line and tissue). We used primers located in PrP exon 2 and PLS3 exon 1 to specifically detect the transgenic expression of human PLS3 from PrP:PLS3. Mouse Pls was specifically amplified using a forward primer that was unique to mouse Pls. The highest level of *PLS3* expression was found in line PLS-14 which showed a 300-fold increase in total spinal cord samples.

### Expression of *PLS3* in motor neurons

**Fig 4:**
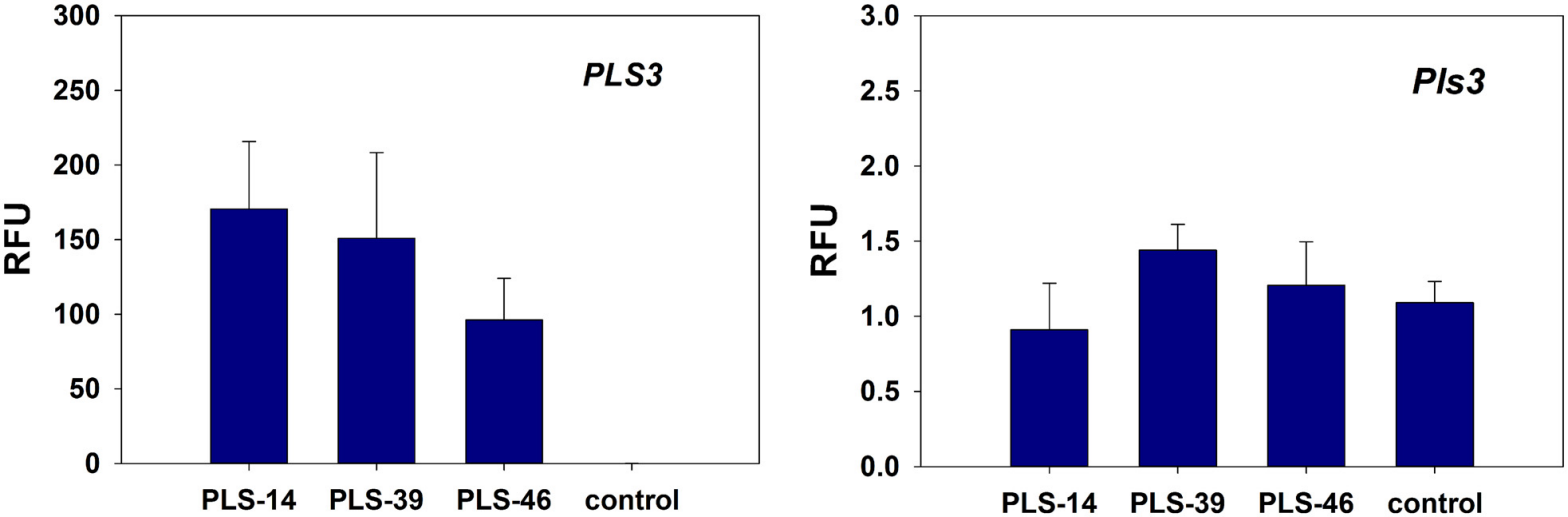
Expression of PLS3 and endogenous mouse Pls3 in LCM isolated motor neurons. Plastin expression was measured by quantitative RT-ddPCR in the motor neurons isolated from lumbar spinal cord tissue at P10 for each transgenic line (A) PLS3 expression is greatest in transgenic line PLS-14. (PLS-14: 170.5±45.3 p<0.05 vs. control, t-test, PLS-39: 151.0±57.7, PLS-46: 96.2±27.8, control: 0±0.0 RFU). Only PLS-14 expression was statistically different from control thus we pursued this line for protein analysis. PLS3 expression was not detected in the non-transgenic control motor neurons indicating the specificity of our primers. (B) Expression of mouse Pls is unchanged in the transgenic PrP:PLS3 lines when compared to a non-transgenic control. (PLS-14: 0.9±0.3, PLS-39: 1.4±0.2, PLS-46: 1.2±0.3, control: 1.1±0.1 RFU, not statistically significant from control.) Note that overexpression of human PLS3 is more than 100 fold greater than the amount of mouse Pls3 expression in the motor neuron. These results are similar to the expression assayed by qPCR in total spinal cord samples. (n = 3 mice for each transgenic line and control). RFU is defined as Relative Fluorescent Units.

To ensure that PrP:PLS3 was indeed expressed in neurons we sectioned P10 lumbar spinal cord (L3-L5) from each transgenic line. The motor neurons were isolated by laser capture microdissection (LCM) and RNA was extracted. One round of aRNA amplification (Arcturus) was followed by quantitative PCR using droplet digital PCR (ddPCR, Bio-Rad). The amount of PLS3 expression detected in the motor neuron is more than 100x greater than the endogenous mouse Pls3 expression for each transgenic line examined (Fig 4A). The level of mouse Pls3 expression for each transgenic line was no different from that of a non-transgenic age matched control (Fig 4B).

### Total Plastin protein expression in spinal cord tissue

**Fig 5:**
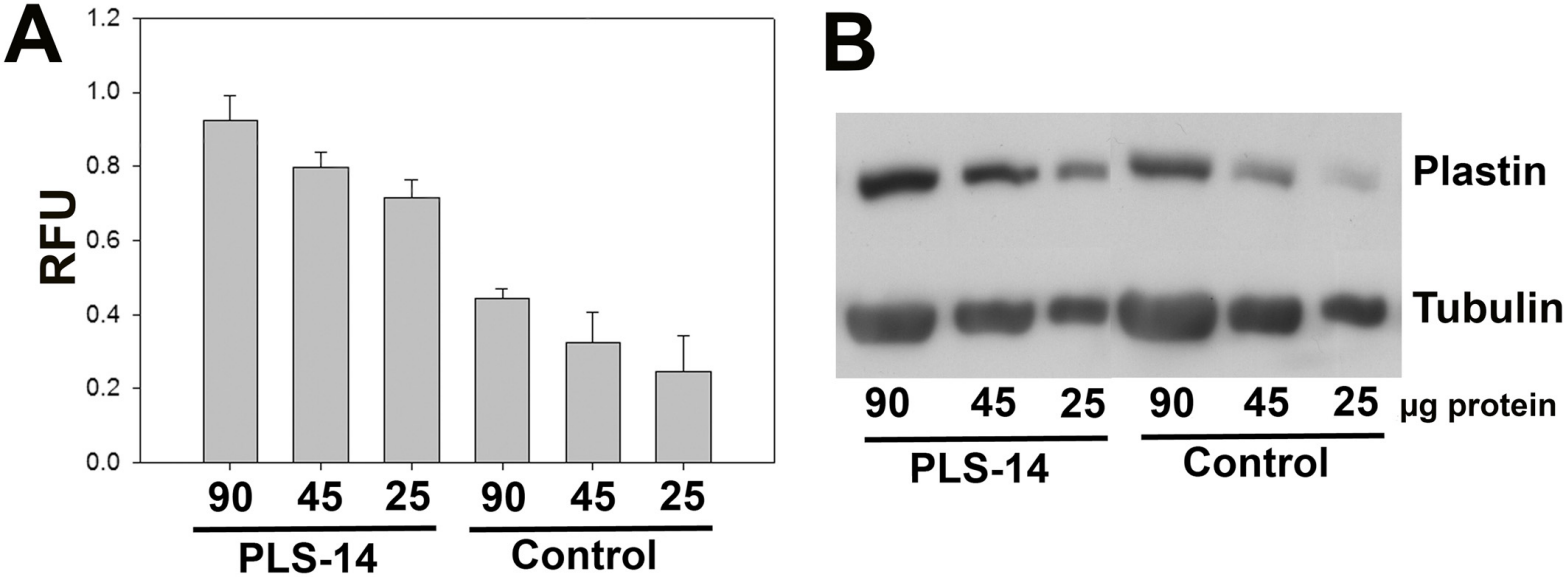
Total PLS3 Protein Expression in Spinal Cord tissue. Total Plastin3 (PLS3 and mouse Pls) protein levels were measured in spinal cord extracts from P10 mice. While the level of PLS3 transcript is 100-fold increased, we found only a 2-fold increase in PLS3 protein in spinal cord samples. (A) Quantification was performed on 90μg, 45μg and 25μg of total protein with 3 independent samples for each protein concentration. A dilution series of total protein was loaded for each sample and the relative mean was determined by dividing the area under PLS3 peak by the area under the tubulin peak. (B) A representative 90μg, 45μg and 25μg serial dilution of one of the PrP:PLS3 samples from line PLS-14 and a non-transgenic control from the Western blot is shown. A total of 3 male mice were assayed for each protein concentration. All concentrations of PrP:PLS3 showed a statistically significant increase in PLS3 when compared to non-transgenic controls (t value 5.204 (90μg), 5.097 (45μg) and 5.086 (25μg) p<0.005) as determined by a one-way ANOVA.

We next examined the expression of total PLS3 protein. The Plastin antibody used was first tested to ensure that it reacted with PLS3 protein at the correct size (~70kD). MN-1 cells were transiently transfected with the PrP:PLS3 construct and the approximately 70kd PLS3 protein was detected by Western blot on transfected cells. In the case of transfected MN-1 cells a marked increase in total PLS3 expression was observed at the protein level. Western blot analysis of total spinal cord from P10 mice expressing PLS3 is shown in Fig 5. Despite a 100-fold increase in *PLS3* mRNA expression, the increase in total PLS3 protein levels was only 2 fold when compared to non-transgenic animals. There was a significant increase in PLS3 protein in the PLS-14 line (p<0.005) (Fig 5A). This result is similar to the findings of Ackerman et al in which the PLS3 transgene was tagged and therefore more easily detected [50]. However the levels indicated for total PLS3 and mouse Pls3 combined are similar to Figure 7 in Ackerman et al. [50]. Thus *PLS3* mRNA is dramatically increased in the spinal cord but post-translational regulation mechanisms present in the mouse limit the level of PLS3 protein that can be obtained. Any regulation of the PLS3 protein is important to consider in determining if *PLS3* expression alters SMA.

### Effect of PLS3 expression on SMA phenotype

**Fig 6:**
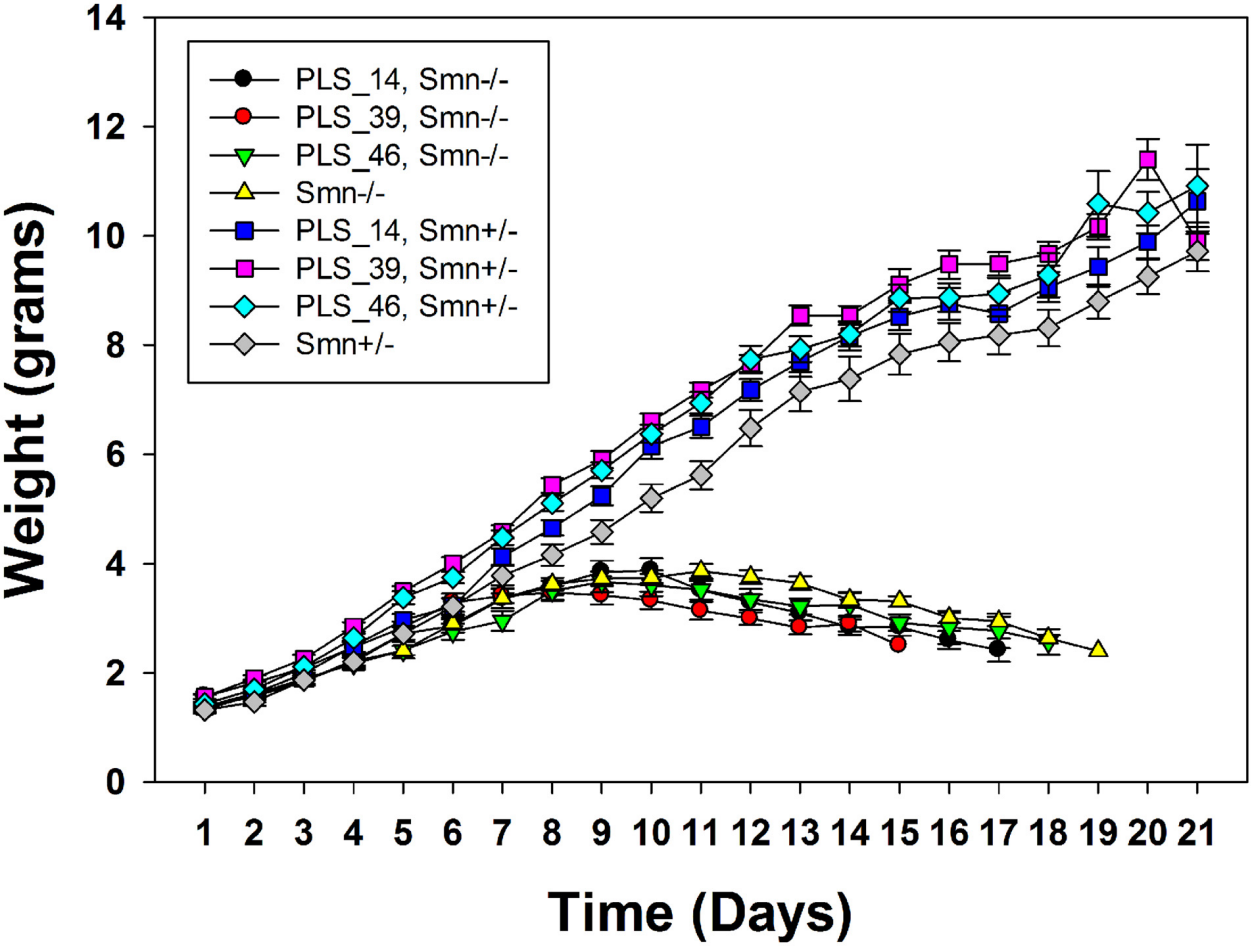
Weight of each PrP:PLS3 transgenic line in the presence and absence of mouse *Smn*. Mice were weighted every day until weaning at 21 days of age. Each of the three PrP:PLS3 transgenic lines (PLS-14^+/-^, *SMN2* ^*+/+*^; *Smn* ^*-/-*^; Δ7SMN^+/+^, n = 22, average max. weight 3.9g), (PLS-39^+/+^, *SMN2* ^*+/+*^; *Smn* ^*-/-*^; Δ7SMN^+/+^, n = 20, average max. weight 3.5g), (PLS-46+/+, *SMN2+/+; Smn* ^*-/-*^; Δ7SMN+/+, n = 32 average max. weight 3.7g) weigh slightly less than Δ7 SMA mice (*SMN2* ^*+/+*^; *Smn* ^*-/-*^; Δ7SMN^+/+^, n = 11, average max. weight 4.0g). There is no statistical difference in weight between SMA mice (*Smn* ^*-/-*^ *)* with or without the transgene.

We measured the weight of PrP:PLS3 mice to determine if overexpression of PLS3 in neurons increased the weight of Δ7 SMA the mouse. We found that the weight is not increased in three PrP:PLS3 transgenic lines in the Δ7 SMA mouse (Fig 6). The weight of each PrP:PLS3 transgenic line in the presence and absence of mouse *Smn* was measured daily until weaning at 21 days of age. The three PrP:PLS3 transgenic lines (PLS-14^+/-^, *SMN2* ^*+/+*^; *Smn* ^*-/-*^; Δ7SMN^+/+^, n = 22, average max. weight 3.9g), (PLS39^+/+^, *SMN2* ^*+/+*^; *Smn* ^*-/-*^; Δ7SMN^+/+^, n = 20, average max. weight 3.5g), (PLS-46+/+, *SMN2+/+; Smn* ^*-/-*^; Δ7SMN+/+, n = 32 average max. weight 3.7g) weigh slightly less than Δ7 SMA mice (*SMN2* ^*+/+*^; *Smn* ^*-/-*^; Δ7SMN^+/+^, n = 11, average max. weight 4.0g). There is no statistical difference in weight between Δ7 SMA mice or any of the transgenic PrP:PLS3 expressing lines as determined by the CompareGrowthCurve function found in the R-Package (Statmod).

**Fig 7:**
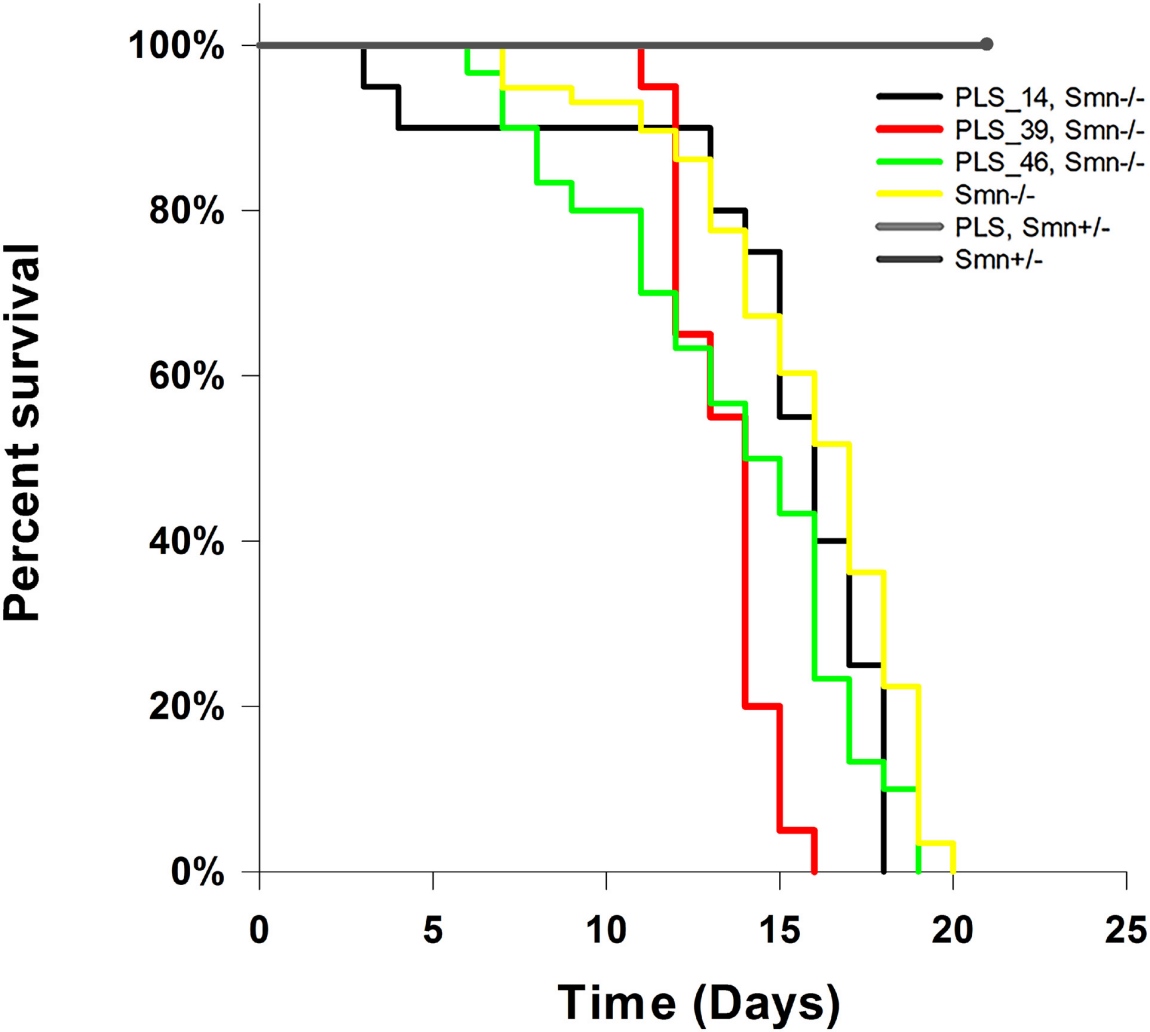
Survival is not increased in PrP:PLS3, Δ7SMA mice. The Kaplan–Meier survival curve for three PrP:PLS3 transgenic lines (PLS-14^+/-^, *SMN2* ^*+/+*^; *Smn* ^*-/-*^; Δ7SMN^+/+^, n = 20), (PLS-39^+/+^, *SMN2* ^*+/+*^; *Smn* ^*-/-*^; Δ7SMN^+/+^, n = 20), (PLS-46^+/+^, *SMN2* ^*+/+*^; *Smn* ^*-/-*^; Δ7SMN^+/+^, n = 30) and SMA (*SMN2* ^*+/+*^; *Smn* ^*-/-*^; Δ7SMN^+/+^, n = 58) is shown. The median survival of PLS-14; Smn^-/-^ (14.8±0.9 days, p = 33) and PLS-46; Smn^-/-^ (13.6± 0.7 days, p = 0.05) was not statistically different from that of Δ7 SMA mice (*Smn* ^*-/-*^ *)* (15.7± 0.4 days). The survival of PLS-39, Smn^-/-^ (13.4± 0.3 days, p<0.01) was statistically different from Δ7 SMA mice (*Smn* ^*-/-*^ *)* (15.7± 0.4 days) as the PLS-39 mice died on average 2 days before the SMA mice (Holm-Sidak pairwise comparison).

To determine if survival of the Δ7 SMA mouse is improved upon overexpression of PLS3 in neurons we monitored survival. Survival is not increased in three PrP:PLS3 transgenic lines in the Δ7 SMA mouse (Fig 7). The Kaplan–Meier survival curve for PLS3 transgenic lines: (PLS-14^+/-^, *SMN2* ^*+/+*^; *Smn* ^*-/-*^; Δ7SMN^+/+^, n = 20), (PLS-39^+/+^, *SMN2* ^*+/+*^; *Smn* ^*-/-*^; Δ7SMN^+/+^, n = 20), (PLS-46+/+, *SMN2+/+; Smn* ^*-/-*^; Δ7SMN+/+, n = 30), and SMA (*SMN2* ^*+/+*^; *Smn* ^*-/-*^; Δ7SMN^+/+^, n = 58). The median survival of PLS-14; Smn^-/-^ (14.8±0.9 days), PLS-46; Smn^-/-^ (13.6± 0.7days) and Δ7 SMA mice (*Smn* ^*-/-*^ *)* (15.7± 0.4 days) were statistically different from controls (log-rank p<0.001). Survival of and Δ7 SMA mice (*Smn* ^*-/-*^ *)* (15.7± 0.4 days) was not statistically different from PLS-14; *Smn* ^*-/-*^ (14.8±0.9 days), or PLS-46; *Smn* ^*-/-*^ (13.6±0.7days) (p<0.001, Holm-Sidak pairwise comparison). PLS-39; *Smn* ^*-/-*^ (13.4±0.3 days) mice died on average 2 days before SMA controls.

### Electrophysiology of PLS3 SMA mice

We and others have previously shown that early stages of SMA disease pathogenesis are characterized by functional abnormalities of the neuromuscular junction (NMJ) [46, 51–53]. It has recently been reported that PLS3 expression rescues function of the NMJ in mice with SMA [50]. In order to determine whether expression of PLS3 rescues functional NMJ abnormalities we examined the physiology of NMJs in the tibialis anterior muscle of mice on P10 to P13 as previously described [46, 51]. Each PrP:PLS3; Δ7SMA mouse was compared to an age-matched littermate that was studied on the same day.

**Fig 8:**
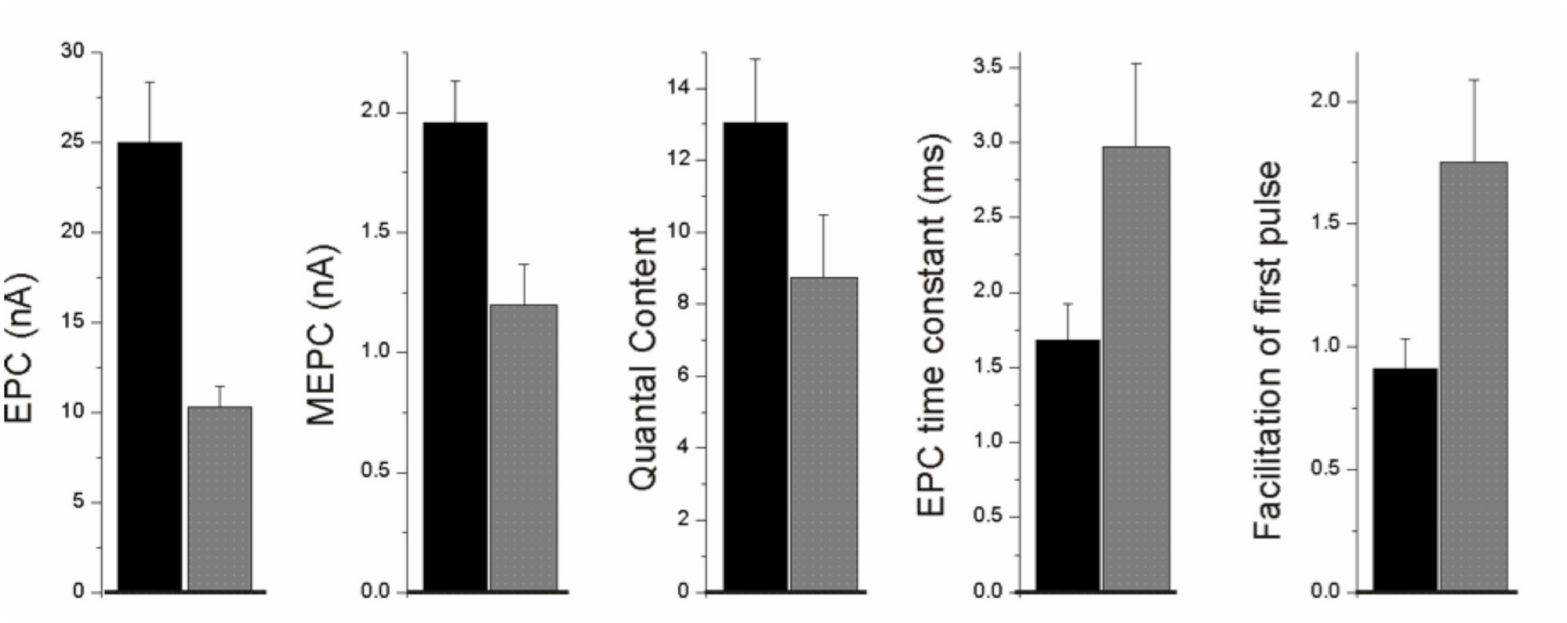
Electrophysiology of PrP:PLS3 transgenic mice in the absence of *Smn*. Analysis of the PLS-14 line in the presence and absence of Smn is compared (PLS-14^+/-^, *SMN2* ^*+/+*^; *Smn* ^*+/-*^; Δ7SMN^+/+^, compared to PLS-14^+/-^, *SMN2* ^*+/+*^; *Smn* ^*-/-*^; Δ7SMN^+/+^). Shown are bar graphs of endplate current (EPC), miniature endplate current (MEPC), quantal content, endplate current time constant (EPC time constant), and the degree of facilitation of the first EPC compared to the 10^th^ EPC during the first 10 pulses of a 50 Hz simulation. All of the deficits in plastin SMA mice are similar in magnitude to the deficits we previously reported in the same line of Δ7 SMA mice. Control littermates (PLS-14^+/-^; *SMN2* ^*+/+*^; *Smn* ^*+/-*^; Δ7SMN^+/+^) = black bars, plastin SMA (PLS-14^+/-^; *SMN2* ^*+/+*^; *Smn* ^*-/-*^; Δ7SMN^+/+^) mice = grey bars. Error bars represent SEM. *n = 4* mice and greater than 40 endplates for all data.

The most dramatic abnormality in SMA is a 60% reduction in endplate current (EPC) amplitude, which is determined by both the number of synaptic vesicles released following nerve stimulation (quantal content) and the amplitude of the muscle response to the transmitter released from a single vesicle (quantal amplitude) [46, 51]. When plastin SMA mice were compared to their unaffected littermates they had a 60% reduction in EPC amplitude (p <0.05, Fig 7) that was very similar to the reduction we found previously in the same line of SMA mice at P10-P14 [46, 51]. The reduction in endplate current amplitude was due to both reduction in quantal content and quantal amplitude (Fig 8) with the magnitude of reduction of both parameters similar to what we found previously in SMA mice at P10-P14 [51].

Previously, we found an increase in MEPC and EPC time constants that was likely due to prolonged expression of embryonic acetylcholine receptors (AChRs) [46, 51]. A similar increase in EPC time constant was present in PrP:PLS3 SMA mice (p <0.05, Fig 8). Finally, we and others previously found that a reduction in the probability of synaptic vesicle release as shown by increased facilitation during repetitive stimulation was a likely contributor to reduced quantal content in SMA NMJs [46, 51, 53]. A similar increase in facilitation was present in PrP:PLS3 SMA mice relative to control littermates (p <0.05, Fig 8).

## Discussion

The overexpression of PLS3 has been suggested to modify the SMA phenotype [36]. In particular, it has been suggested to act as a female specific modifier thus overexpression of PLS3 would only alter female SMA patients. This finding was reported after the identification of higher PLS3 expression in lymphoblasts of the less severely affected SMA individual of siblings with identical haplotypes but variant phenotype. However, an increase in PLS3 expression does not occur in all haploidentical cases. Thus it was reported that PLS3 is a female specific modifier of SMA phenotype that is not always penetrant [36]. A close examination of the 6 pedigrees studied reveals that in all but one example (family #34), the severely affected case was male [35, 36]. Thus low PLS3 expression would not be predicted to have any impact on these individuals anyway. In essence, the initial evidence for PLS3 modifying the SMA phenotype comes down to one family where the two mildly affected female patient showed a one fold (BW279) and 1.6 fold (BW283) increase in *PLS3* transcript compared to their more severe sister (BW280) [36]. In a separate study female patients with the more severe phenotype showed high PLS3 expression compared to their less severe female siblings [54]. Thus it does not seem PLS3 expression can always modify SMA in females and it is unclear why this modification would be partially penetrant and female specific. Lastly no clear insight into how increased PLS3 expression occurs has been presented. Does it occur due to an alteration of regulatory sequence at the PLS3 locus, alteration of methylation at the PLS3 locus alteration of a transregulator of PLS3 expression or altered escape from X inactivation at that loci. In the latter case it can be noted that PLS3 has been reported to undergo X inactivation [55] and it is hard to see how this would specifically give rise to high PLS3 expression in certain individuals. Furthermore males do show high *PLS3* expression but modification is not reported to occur in this case (family #800, individual LN421 found in Oprea, et al.) [36]. Regardless it is important to address the issue of how PLS3 is activated as well as why *PLS3* expression is only believed to operate in certain individuals.

In a subsequent study, Stratigopoulos et al. [56] found no difference in *PLS3* expression in 47 female SMA patients when all ages, SMA types or *SMN2* copy number were compared. An inverse correlation between *PLS3* expression and SMA severity was only identified when females were grouped by age (pre and post pubescence). No change in *PLS3* expression was identified in males grouped by SMA severity or *SMN2* copy number. *PLS3* levels were found to be 50% lower in older males. Finally, expression of *PLS3* did not correlate with the functional measures of CMAP or MUNE in males or females [56].

Recently, Yanyan et al. found higher levels of PLS3 in type 3 female SMA children (over the age of 3) compared to type 2 female children [57]. Yet the level of *PLS3* expression was always higher in females than in males and correlated positively with *SMN2* copy number. The level of *PLS3* was higher in SMA patients 3 to 12 years of age compared to healthy controls. Thus it is suggested that *PLS3* may be playing some compensatory role in SMA, however levels of *PLS3* were highest in healthy controls under age of 3. *PLS3* is unlikely to be useful as a biomarker due to the alteration of expression in blood with patient age and sex [37].

Although PLS3 mRNA is elevated in lymphoblasts at the mRNA level there are differences in the reports of protein expression. Opera et al. [36] reported altered PLS3 protein levels whereas Bernal et al. [54] found that PLS3 protein levels where not detectable in lymphoblasts and not significantly altered in fibroblasts of patients that had different mRNA levels of *PLS3*. Similarly, in our study we observed only a 2 fold increase in PLS3 protein in transgenic mice heavily overexpressing (up to 300 fold higher) *PLS3* mRNA indicating the likely occurrence of posttranslational regulation of PLS3 expression. Hao et al. [58] have reported in zebrafish that reduction of SMN resulted in reduced PLS3 protein levels whereas in the mouse Ackerman et al. [50] found that SMN levels did not alter PLS3 levels. Other studies have shown that *PLS3* levels are increased under various conditions. For instance cisplatin-resistant human bladder, prostatic, and head and neck cancer cell lines express high levels of *PLS3* when compared to cisplatin-sensitive cells [59]. High *PLS3* levels have also been found in Sezary Syndrome patients and this was associated with loss of CD26. In addition, *PLS3* positive cells showed hypomethylation of the *PLS3* CpG island at sites 95–99 [60, 61]. Interestingly, the polymorphism SNP PLS3 rs871773 T allele is associated with a higher protein expression of the *PLS3* gene in colon cancer and an increased risk of recurrence of colon cancer [62]. If PLS3 does alter severity of SMA, defining the role of both *PLS3* rs871773 and the hypomethylation of sites 95–99 is important as it gives a mechanism of PLS3 activation and may even result in a DNA marker that could be followed in patient material. However, this does not explain why increased *PLS3* expression only modifies female SMA patients and is often non-penetrant. Indeed our results show no marked alteration of SMA phenotype in mice with a 100-fold increase in mRNA expression of *PLS3*. The studies we present here do not support a role for *PLS3* in SMA. Moreover, the lack of penetrance in modifying the phenotype in males, as well as certain female cases, is difficult to reconcile.

Previously, overexpression of PLS3-V5, which contains an amino terminal tag, was reported to improve the Taiwanese model of SMA [50, 63] but only very slightly and under specific conditions [50]. No improvement of survival of the Taiwanese 2 copy *SMN2* mice was seen on a C57BL/6 background with overexpression of PLS3-V5 and only marginal improvement of muscle fiber size and connectivity of the NMJ. In a F1 mixed background of FVB/N and C57BL/6 the mean survival rate was increased by 2 days and the maximum survival was not increased [50]. In our experience with the Δ7 SMA mice this kind of survival increase is not significant and can vary between tests. In essence, the differences in survival between the current study and that of Ackerman et al. are minimal and we suggest there is no significant improvement in survival of Δ7 SMA mice with overexpression of *PLS3*. Alternatively, the modest increase in survival of 2 days in the mixed background Taiwanese SMA mouse model could be due to a neuroprotective effect of PLS3. A slight increase in survival was also observed in the Taiwanese SMA mice upon administration of neuroprotective factors *IGF-1* [64], *cardiotrophin-1* [65], and *Bcl-xL* [66].

In our study as well as that of Ackerman et al., electrophysiology studies of the function of the NMJ were performed. Ackerman et al. reported a small improvement in the time constant of the endplate potential and quantal content [50]. However this was only on a mixed background and is unlikely to have a major impact on NMJ function. We did not find evidence to suggest significant improvement in either parameter. There is no evidence to suggest that expression of *PLS3* improved any of the pre- and postsynaptic physiologic deficits at the neuromuscular junction in our study of Δ7 SMA mice. One difference between our study and the previous study is that we used voltage clamp of muscle fibers to directly measure synaptic currents whereas the study by Ackermann et al. measured endplate potentials. Endplate potentials can be affected by changes in muscle fiber properties (fiber size and specific membrane resistance) that are unrelated to synaptic function. These differences might account for the difference in findings relating to time constant, however, it seems unlikely that a difference in muscle fiber property could account for the difference between the two studies on quantal content. We cannot rule out that overexpression of *PLS3* has a very modest effect on synaptic function that would be picked up with study of more mice. Driving expression of human PLS3 in motor neurons rescued the NMJ defects and motor function in zygotic zebrafish *smn* mutants suggesting that under low conditions of SMN, PLS3 can indeed benefit vertebrate motor neurons [58]

In conclusion, we have shown in the Δ7 mouse model of SMA no beneficial effects of *PLS3* overexpression in the neuron. This is also consistent with the study of Bowerman et al. [67] using a milder model of SMA where loss of Profilin results in increased *PLS3* expression but no modification of the SMA phenotype [67]. A puzzling feature of all the reports of PLS3 modification is that the effect is proposed to be sex-specific and partially-penetrant. To date, this hypothesis has not been replicated in any animal studies and is not explained by *PLS3*’s location on the X chromosome because a transgene on an autosome will not be subjected to inactivation. It is clear that there are males in the population that express *PLS3* but this does not modify the SMA phenotype in humans. Why would this be the case? We suggest that modifiers of SMA need to be revisited in the human population in discordant sibling pairs. A genetic modifier that has a DNA change or a solid mechanistic base as to why altered plastin expression occurs can be studied in these individuals. In this case it would be preferable to study haploidentical pairs of discordant type 1 and type 2, or type 2 and type 3 siblings. The genetic modifier will not be present in any severe SMA type 1 patient therefore type 1 patient DNA can be used to exclude false modifiers. Indeed there are SNPs and methylation changes associated with altered *PLS3* expression that could be investigated in SMA. Currently *PLS3* can be viewed as a candidate modifier where an understanding of mechanism of activation, and DNA changes associated with increased expression is not understood, nor why modification only occurs under certain circumstances. However an alternative explanation is that *PLS3* is in fact not the critical modifier of SMA phenotype. Thus studies that remain open to the possibility of defining alternate modifiers in SMA are of critical importance.
